# A Case of Acute Pancreatitis in Hemolysis, Elevated Liver Enzymes, and Low Platelets Syndrome

**DOI:** 10.7759/cureus.2877

**Published:** 2018-06-25

**Authors:** Manuel Pereira Herrera, Joshua B Oaks, Jeffrey Brensilver

**Affiliations:** 1 Medicine, Overlook Medical Center, Summit, USA; 2 Internal Medicine, Overlook Medical Center, Summit, USA

**Keywords:** acute pancreatitis, pregnancy, hellp syndrome, preeclampsia

## Abstract

We present a case of hemolysis, elevated liver enzymes, and low platelets (HELLP) syndrome complicated by acute pancreatitis that responded favorably to conservative measures. The microvascular abnormalities and heightened inflammatory state present in HELLP syndrome and severe preeclampsia might be responsible for pancreatic ischemia or cytokine-induced pancreatic damage, which could result in acute pancreatitis.

## Introduction

Acute pancreatitis is a condition with acute inflammation and in some cases necrosis of the pancreas. Etiologies for pancreatitis include biliary disease, alcohol, hypertriglyceridemia, iatrogenic causes (surgical or post-endoscopic retrograde cholangiopancreatography), medications, autoimmunity, trauma, and ischemia [1-3].

Hemolysis, elevated liver enzymes, and low platelets (HELLP) syndrome is a condition that belongs to the hypertensive disorders of pregnancy, which include preeclampsia/eclampsia [4]. HELLP syndrome has been associated with severe complications, such as placental abruption, disseminated intravascular coagulation (DIC), pulmonary and cerebral edema, and stroke, among others [1,4-5]. Substantial alterations in the microcirculation have been observed in HELLP syndrome, likely due to microangiopathy and vasospasm [6], the combination of which is thought to contribute to end-organ damage. HELLP syndrome and severe preeclampsia have been reported as etiological factors of pancreatitis [7-11], but this association is yet to be fully accepted. We present a case of pancreatitis in the setting of HELLP syndrome and a review of the literature.

## Case presentation

A 32-year-old female, gravida 2 para 1 at gestational age of 36 weeks and two days, presented to the Overlook Medical Center at 2:57 am complaining of nausea and contractions, which had started earlier in the night and worsened. Prenatal records were unavailable. Her previous pregnancy was complicated by premature rupture of membranes, and she delivered vaginally a healthy infant at 36 weeks of gestational age.

During intake, the patient sat upright, appearing uncomfortable, and complained of epigastric pain and vomiting. The physical examination was remarkable for a blood pressure of 202/101 mmHg. Initial laboratory results were significant for the following: white blood cell count (WBC) 13.6 x 10^3^/µL, urine protein of 300 mg/dL, hemoglobin (HGB) 15.6 g/dL, platelets (PLT) 182 x 10^3^/µL, lipase 200 IU/L, total bilirubin (T bili) 0.2 mg/dL, aspartate transaminase (AST) 56 IU/L, alanine transaminase (ALT) 40 IU/L, alkaline phosphatase (ALP) 162 IU/L, and albumin 2.3 g/dL.

Upon a diagnosis of severe preeclampsia, the patient was started on intravenous (IV) magnesium sulfate for seizure prophylaxis at 2 g/h and was given IV labetalol to control blood pressure. The fetal non-stress test was reactive and category 1. The patient was scheduled for emergent cesarean section, which was carried out without complications three hours after admission, resulting in the delivery of a vigorous 1.645 kg male infant, with appearance, pulse, grimace, activity, respiration (APGAR) scores of 8/9 at one and five minutes, respectively. The placenta was delivered complete, and the blood loss during surgery was 680 mL. In the recovery room, the patient continued with high blood pressure values in the 190s/110s mmHg and was given boluses of 10 mg of hydralazine IV in addition to IV labetalol.

Two hours post-operation (post-op), the patient reported severe right upper quadrant pain with worsening nausea and vomiting. Laboratory data at that time revealed up-trending liver enzymes (AST of 1054, ALT of 687, ALP of 134 IU/L), a T bili that had risen but was still in the normal range of 0.8 mg/dL, worsening of her hypoalbuminemia at 1.7 g/dL, anemia of 11.2 g/dL, thrombocytopenia of 59 x10^3^/µL, and worsening leukocytosis with neutrophilia (19.9 x10^3^/µL and 87.3%). Prior to this point, the patient had received 1.6 L of IV fluids. The international normalized ratio (INR) was slightly elevated at 1.19, and the fibrinogen was decreased at 192 mg/dL, with fibrin degradation products ≥20 µg/mL. The patient was then transferred to the intensive care unit (ICU) with the diagnosis of HELLP syndrome. A chest x-ray to rule out pulmonary edema reported no significant findings.

**Figure 1:**
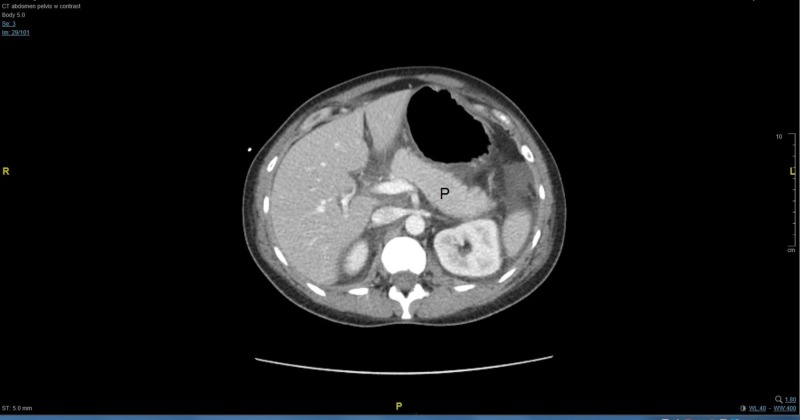
Abdominal computed tomography (CT) scan with contrast highlighting the pancreas P:pancreas

Six hours post-op, the patient still complained of right upper quadrant pain and nausea, but these were slightly improving. Her physical exam was remarkable for decreased breath sounds bilaterally at the lung bases and diffuse abdominal tenderness, which was more prominent on the left side. Subsequent laboratory values reported worsening transaminitis with AST of 1332, ALT of 663, ALP of 146 IU/L, increased T bili of 1.1 mg/dL, WBC of 20.5 x10^3^/µL, and worsening PLT of 38 x10^3^/µL. An elevated lactate dehydrogenase (LDH) of 1881 IU/L and a decreased haptoglobin of <8 mg/dL pointed to a hemolytic process consistent with HELLP syndrome. Although the patient was afebrile, empiric antibiotic coverage with IV cefazolin 2 g every eight hours was initiated out of concern for endometritis given her recent delivery. Her fibrinogen levels had improved, however, to 228 mg/dL. Magnesium levels were monitored, and the magnesium infusion was adjusted accordingly. Blood pressure levels showed signs of better control, with values in the 150s/105s mmHg, and she was maintained on an IV labetalol infusion. A chest x-ray was ordered, and was reported clear. Worsening thrombocytopenia and liver function tests prompted an abdominal computed tomography (CT) scan, which revealed a small volume of complex abdominal and pelvic ascites, mild bilateral pleural effusions and bibasilar atelectasis, and no evidence of subcapsular hematoma, liver, or gallstone pathology. Upon further review of the CT scan, it was felt that the pancreas appeared prominent, which prompted further workup. We include an image of the CT scan below in Figure 1.

Twelve hours post-op, the patient had improved blood pressure values in the 130s/90s mmHg. Pancreatitis was confirmed with a lipase of 2365 IU/L, which had risen from 200 IU/L on admission. Laboratory findings also showed increased lactic acid of 2.9 mmol/L, bicarbonate (HCO_3_-) of 22 mEq/L, worsening anemia with HGB of 10.1 g/dL, down-trending liver enzymes (AST of 944, ALT of 536 IU/L), and improved leukocytosis (WBC of 17.3 x10^3^/µL). The labetalol infusion was discontinued at this point, given her lower blood pressure values. Labetalol, as needed, was ordered to maintain a blood pressure goal of below 150s/110s mmHg.

In the morning of the second day after admission, the patient´s blood pressure improved to 145-118 /92-79 mmHg, her right upper quadrant pain was reduced, and her physical exam revealed decreased abdominal tenderness. Laboratory values showed down-trending transaminitis (AST of 573, ALT of 415, ALP of 111 IU/L), and decreasing LDH of 1030 IU/L. The anemia and thrombocytopenia had worsened to HGB of 9 g/dL and PLT of 36 x10^3^/µL, with increasing WBC 15.6 x10^3^/µL. The lipase level had increased to 2509 IU/L together with the lactic acid 3.9 mmol/L, yet her HCO_3_- level remained normal at 23 mEq/L. On the evening of hospital day 2, she started tolerating clear liquids with only mild nausea. Laboratory results eventually showed normalization of the lipase at 186 IU/L, and her liver enzyme levels continued trending downward, along with trends toward normalization of her other laboratory values.

On the third day after admission, the patient maintained normal blood pressure values (110s/80s mmHg) with oral labetalol. Her abdominal pain continued decreasing and her nausea stopped. She remained on the magnesium sulfate infusion and was transferred from the ICU back to the obstetrics floor. The patient continued to improve on her fourth day, with decreasing liver enzymes (AST of 84, ALT of 113, ALP of 99 IU/L), increasing platelets (PLT 80 x10^3^/µL), and decreasing WBC at 13.3 x10^3^/µL, but her HGB dropped to 7.9 g/dL. The patient was not transfused, as she was hemodynamically stable. Importantly, her creatinine levels remained below 1.1 mg/dL throughout her hospitalization. The patient was subsequently discharged on the fifth day after admission, with improved HGB of 8.5 g/dL, PLT of 127 x10^3^/µL, WBC of 11.7 x10^3^/µL, and normalization of her transaminitis.

## Discussion

Preeclampsia is defined as a new onset of hypertension in pregnancy (≥ 140/90 mmHg after 20 weeks of gestation) together with proteinuria (≥ 300 mg/day or a protein/creatinine spot urine ratio of at least 0.3, each measured as mg/dL) [12]. Severe preeclampsia requires the presence of any of the following features: hypertension ≥160/110 mm Hg, thrombocytopenia <100,000/µL, elevated liver enzymes above twice the upper limit of normal, severe persistent right upper quadrant or epigastric pain, serum creatinine >1.1 mg/dL or doubling of serum creatinine, pulmonary edema, or new-onset cerebral or visual disturbances [12]. HELLP syndrome is seen as a particularly serious complication of severe preeclampsia [1,5]. There are two principal diagnostic schemes for HELLP syndrome. The Tennessee classification includes the following: 1) evidence of microangiopathic hemolytic anemia by the presence of schistocytes in the blood smear, low serum haptoglobin (< 1g/L - <0.4 g/L), and elevated LDH levels; 2) AST > 70 IU/L, LDH > 600 IU/L or total bilirubin > 1.2 mg/dL; and 3) a platelet count below 100,000/µL. The Mississippi classification further stratifies the syndrome based on the platelet nadir [1]. Complete HELLP syndrome is diagnosed when the three components are present, while partial or incomplete HELLP syndrome requires only one or two components. Patients with partial HELLP syndrome tend to develop fewer complications than those with the complete form [5].

The incidence of preeclampsia in pregnancy is reported to be 3%-5% [13], while HELLP syndrome complicates about 0.2%-0.9% of all pregnancies [1,5]. HELLP tends to occur in about 10% to 20% of cases with severe preeclampsia, and it appears 30% of the time after delivery [5]. Several mechanisms contribute to the derangements seen in HELLP syndrome. The interaction of maternal immune and vascular endothelial cells with syncytiotrophoblast particles and other placental products triggers an intense inflammatory response, greater than that seen in preeclampsia, which activates the maternal endothelium, promotes platelet aggregation, activates the coagulation cascade, and leads to thrombotic microangiopathy with the resulting hemolytic anemia [1].

The hepatic injury in HELLP is mediated by a placental product, the Fas-ligand (FasL or CD95L), which triggers the synthesis of tumor necrosis factor (TNF) - α, resulting in hepatocyte apoptosis and necrosis. The hepatotoxicity of FasL is compounded by ischemic liver damage resulting from restricted portal blood flow, likely related to microangiopathy [1]. Ischemia to important vascular beds may be a consequence of HELLP syndrome and, to a lesser extent, of preeclampsia. Substantial distributive microcirculatory derangements (reduced percentage of small-vessels perfused, reduced functional capillary density, and reduced total capillary density) have been observed in the sublingual microcirculation in patients with preeclampsia compared with healthy pregnant controls. These microvascular alterations were even more severe in HELLP syndrome and were incompletely reversed during the early post-delivery period [6].

Some well-established complications of HELLP syndrome include subcapsular liver hematoma, hepatic rupture, abruptio placentae, DIC with subsequent severe postpartum bleeding, wound hematoma and infection following cesarean section, pulmonary edema, and cerebral infarction, hemorrhage, or edema complicated by brain herniation [1,4-5]. Renal failure, likely secondary to microangiopathy and glomerular endotheliosis, can also be seen in HELLP [1,5]. Retinal detachment, vitreal hemorrhage, and cortical blindness are infrequent complications of HELLP to which DIC may contribute [5]. Several cases of acute pancreatitis believed to be caused by preeclampsia/HELLP syndrome have been reported in the literature, lending credence to the opinion that pancreatitis may be a rare complication associated with these pathologies, particularly with HELLP syndrome [7-11].

The incidence of pancreatitis in pregnancy has been found to be between 0.02%-0.03%, with biliary disease accounting for 67%-100% of all cases [14]. In a recent study, Sang et al. found an incidence rate of 1.23% of acute pancreatitis in pregnant women with severe preeclampsia [8]. Of those women with severe preeclampsia and pancreatitis, 60% were additionally found to have HELLP syndrome [8]. The number of reported cases of acute pancreatitis in the context of severe preeclampsia/HELLP syndrome for which no biliary, pharmacological, or other metabolical etiology has been found has been steadily increasing [7-11]. The mechanisms that may trigger an insult to the pancreas could involve 1) ischemia secondary to the microangiopathy exacerbated by vasospasm of the splanchnic circulation; 2) a trigger of pancreatic inflammation related to the increased inflammatory state present in severe preeclampsia/HELLP; or 3) a combination of these two possibilities.

Pancreatic ischemia of even short duration is a well-established factor for the development of acute pancreatitis [2-3]. In cases of ischemia, elevation of pancreatic enzymes may not be present immediately due to the development of massive pancreatic necrosis [3]. Hojo et al. reported a case of acute pancreatitis and cholecystitis associated with HELLP syndrome in which the serum amylase was not elevated and the diagnosis was made by means of CT imaging showing an enlarged pancreas [11]. Vasospasm of the hepatic arteries has also been observed in HELLP syndrome. These facts, together with the evidence for circulatory compromise of several capillary beds in HELLP [6,11], could indicate that ischemia contributes to the pancreatic injury. In our patient, evidence of hypoperfusion shown by increasing lactic acid levels accompanied an increase in the lipase levels. Similarly, a decrease in the lactic acid levels accompanied a decrease in the lipase levels, raising our suspicion that the pancreatic insult may well be related to ischemia.

Elevated levels of inflammation markers such as C-reactive protein, interleukin (IL) -6, and TNF- α are seen in HELLP syndrome. Activation of complement and high levels of von Willebrand factor are also present [1,15]. Several cytokines elevated in HELLP are able to activate nuclear factor kappa b (NF-kB) [16], and it has been shown that intra-acinar activation of NF-kB can lead to severe acute pancreatitis [17-19]. There is also the possibility that a placental product causing the release of a pancreatotoxic cytokine might be implicated in the pathogenesis of acute pancreatitis in severe preeclampsia/HELLP, mimicking the role of FasL in the liver damage seen in HELLP. Animal studies have reported a novel cytokine, IL-33, as an activator of the proinflammatory pathways in the acinar cells, resulting in acute pancreatitis [20]. Future studies may want to evaluate the role of IL-33 in inducing acute pancreatitis in humans.

## Conclusions

We have presented a case of HELLP syndrome in the post-cesarean section period, complicated by acute pancreatitis. The patient made a full recovery with bowel rest, IV hydration, magnesium sulfate prophylaxis, and careful blood pressure control. It may be important to monitor indicators of tissue hypoperfusion in HELLP syndrome and to rule out possible pancreatitis that may go unnoticed and develop rapidly in these patients.
